# Support for Alcohol Control Policies Among US Alcohol Consumers

**DOI:** 10.1001/jamanetworkopen.2025.35337

**Published:** 2025-10-03

**Authors:** Anna H. Grummon, Carolyn Chelius, Cristina J. Y. Lee, Aline D’Angelo Campos, Noel T. Brewer, Allison J. Lazard, Callie Whitesell, Thomas K. Greenfield, Marissa G. Hall

**Affiliations:** 1 Department of Pediatrics, Stanford University School of Medicine, Palo Alto, California; 2 Department of Health Policy, Stanford University School of Medicine, Stanford, California; 3 Department of Health Behavior, Gillings School of Global Public Health, University of North Carolina at Chapel Hill; 4 Carolina Population Center, University of North Carolina at Chapel Hill; 5 Lineberger Comprehensive Cancer Center, University of North Carolina at Chapel Hill; 6 Department of Nutrition, Gillings School of Global Public Health, University of North Carolina at Chapel Hill; 7 Hussman School of Journalism and Media, University of North Carolina at Chapel Hill; 8 Alcohol Research Group, Public Health Institute, Emeryville, California

## Abstract

**Importance:**

Alcohol control policies are associated with lower alcohol-related disability and death. Policymakers are more likely to adopt policies with higher public support, but it remains unknown which alcohol control policies currently garner the most public support in the US.

**Objective:**

To determine the extent to which US adults who consume alcohol support alcohol control policies and whether support differs by behavioral and demographic characteristics.

**Design, Setting, and Participants:**

This cross-sectional, online survey study of a nationally representative sample of US adults was conducted from September to October 2024. Adults aged 21 years or older who reported drinking at least 1 alcoholic beverage per week during the past 4 weeks were eligible.

**Main Outcomes and Measures:**

The primary outcome was support for alcohol control policies (eg, requiring cancer warnings on alcohol containers or increasing alcohol taxes) measured on a 1 (strongly oppose) to 5 (strongly support) response scale. Associations of behavioral and demographic characteristics with policy support were estimated using average differential effects (ADEs; ie, differences in estimated mean overall support between groups on the 1-5 scale) were calculated.

**Results:**

A total of 1036 participants completed the survey (524 men [weighted percentage, 52%]; mean [SD] age, 49.4 [16.5] years; 138 [weighted percentage, 12%) Hispanic, Latino, or Spanish; 115 [weighted percentage, 11%] Black or African American; 681 [weighted percentage, 65%] White). Approximately one-half supported policies requiring alcohol containers to display cancer warnings (49%; 95% CI, 45% to 53%), drinks per container information (51%; 95% CI, 47% to 55%), and calorie content information (56%; 95% CI, 52%-60%). Likewise, 52% (95% CI, 48% to 56%) supported prohibiting alcohol advertisements on television when children are likely to be watching. Few participants (8% [95% CI, 6% to 11%] to 19% [95% CI, 16% to 23%]) opposed these policies. By contrast, fewer participants supported than opposed policies to lower the blood alcohol content limit for driving, prohibit alcohol sales late at night, and increase taxes on alcohol (range supporting: 16% [95% CI, 13% to 19%] to 25% [95% CI, 22% to 29%]); support was lowest for policies to reduce the number of outlets licensed to sell alcohol (10% [95% CI, 8% to 13%]). Across policies, support was greater among adults who reported drinking less often (ADE = −0.12; 95% CI, −0.23 to −0.02); not binge drinking (ADE = −0.15; 95% CI, −0.26 to −0.04); or reading the current alcohol health warning in the last 30 days (ADE = 0.14; 95% CI, 0.01 to 0.28); and among those who were women (ADE = 0.22; 95% CI, 0.11-0.32); Hispanic, Latino, or Spanish (ADE = 0.16; 95% CI, 0.001-0.33); Democrats (ADE = 0.14; 95% CI, 0.01-0.27); or political independents (ADE = 0.16; 95% CI, 0.02 to 0.29).

**Conclusions and Relevance:**

In this survey study of US adults who consume alcohol, many supported advertising and labeling alcohol control policies, and few opposed these policies. These results suggest that policymakers may wish to pursue advertising and labeling policies as a ground-softening strategy to build support for stronger policies such as increasing taxes or restricting the times and places alcohol can be sold.

## Key Points

**Question:**

To what extent do US adults who consume alcohol support alcohol control policies?

**Findings:**

In this survey study of 1036 US adults who consume alcohol, approximately one-half supported—and few opposed—policies to restrict alcohol advertising to children and to require calorie content information, drinks per container information, and cancer warnings on alcohol containers. Fewer adults supported policies to lower the blood alcohol content limit for driving, increase alcohol taxes, or limit the times or places alcohol can be sold.

**Meaning:**

These findings suggest that among alcohol control policies, public support is highest for advertising restrictions and information provision policies.

## Teaser

This survey study examines the extent to which US adults who consume alcohol support alcohol control policies and whether support differs by behavioral and demographic characteristics.

## Introduction

Every year, alcohol consumption contributes to nearly 180 000 deaths and at least $250 billion in economic costs in the US.^1^ Alcohol control policies are an important strategy for reducing alcohol consumption and alcohol-related deaths.^2,3,4,5^ The US Surgeon General, for example, recently called for the US to adopt cancer warnings for alcohol.^6^ Likewise, the World Health Organization and US Centers for Disease Control and Prevention have recommended policies such as increasing alcohol taxes, limiting how many retailers can sell alcohol in a given area, and limiting the times of day when alcohol can be sold.^4,7^ Jurisdictions that have adopted these and other evidence-based alcohol control policies have lower per capita alcohol consumption^8,9,10,11^ and alcohol-related disability and death,^12,13,14^ even after controlling for economic and demographic characteristics. Researchers estimate that making alcohol control policies just 10% stricter could reduce alcohol-attributable cancer deaths by 8.5%^15^ and alcohol-attributable homicide rates by up to 18%.^16^

The US has considerable room for improvement in adopting evidence-based alcohol control policies,^17^ scoring just 39.8 out of 100 on the Alcohol Control Policy Index, a validated measure of the strength of alcohol control policies.^8^ One important determinant of policy adoption is public support; policymakers are more likely to adopt policies when they perceive that the public supports those policies.^18,19,20,21^ However, current support for alcohol control policies in the US remains largely unknown because no recent nationally representative studies have assessed support for most evidence-based alcohol control policies.^22,23^ Especially important is assessing support among current alcohol consumers, who would be most directly affected by alcohol control policies and therefore may be more likely to oppose adoption of these policies.^24,25,26,27^ Additionally, understanding the associations of behavioral and demographic characteristics with current support for alcohol control policies could help policymakers understand how their constituents would respond to these policies and help advocates identify groups that might champion them. To address these gaps, this study aimed to assess public support for alcohol control policies among US alcohol consumers and identify characteristics associated with support.

## Methods

This survey study was approved by the University of North Carolina institutional review board. Reporting follows the American Association for Public Opinion Research (AAPOR) reporting guideline.

### Participants

Participants were a national probability sample of US adults aged 21 years or older who reported consuming at least 1 alcoholic drink per week during the past 4 weeks. NORC at the University of Chicago recruited study participants from its AmeriSpeak panel, a nationally representative panel with approximately 48 000 panelists across 40 000 households. In contrast with opt-in convenience samples, AmeriSpeak uses complex, 3-stage, probability-based sampling to recruit panelists such that, after weighting, results statistically represent the US population. The weighted household recruitment rate for the study was 26% (eMethods in Supplement 1). We preregistered the study design and analysis plan on AsPredicted.org prior to data collection^28^; deviations from the plan are described below.

### Procedures

We fielded the survey from September to October 2024. After providing electronic informed consent, participants completed an online survey. Participants responded to questions about their support for different alcohol control policies, their behaviors related to drinking (eg, frequency of alcohol consumption), and their demographic characteristics (eg, age and income). Participants received incentives from NORC equivalent to $2.

### Measures

The primary outcome was support for 8 alcohol control policies. To select policies, we identified 4 domains recommended by the World Health Organization as most effective for reducing alcohol-related disability and death^2,3,4^: advertising restrictions, price increases, availability restrictions, and drunk driving policies. Within each domain, we selected 1 to 2 policies that may be legally feasible in the US (ie, because they have been proposed or adopted by some jurisdictions). We additionally assessed support for 3 labeling policies given recent interest in these among advocates and regulators.^6,29,30,31,32^ The 8 policies were (using the wording shown in the survey): prohibiting alcohol advertisements on television when children are more likely to be watching; requiring alcoholic beverages to list the number of calories in the container; requiring alcoholic beverages to list the number of standard drinks in the container; requiring alcoholic beverages to display warnings that alcohol can cause cancer; lowering the blood alcohol content (BAC) at which people can legally drive; prohibiting stores, bars, and restaurants from selling alcohol late at night; increasing taxes on alcoholic beverages; and reducing the number of stores, bars, and restaurants that are licensed to sell alcohol in a given area.

Participants rated their support for each policy using an item adapted from prior studies:^22,33,34^ “For each of the following policies, indicate how much you would support or oppose that policy.” The policies were shown in random order. Response options were strongly oppose (coded as 1), somewhat oppose (2), neither oppose nor support (3), somewhat support (4), and strongly support (5). We categorized participants as opposing each policy if they somewhat or strongly opposed it, neutral if they neither opposed nor supported it, and supporting if they somewhat or strongly supported it. We additionally assessed overall support for alcohol control policies by averaging responses across all 8 policies (Cronbach α = 0.74).^34,35^

Participants also responded to questions about behavioral and demographic characteristics that have been associated with support for alcohol control policies in prior studies or that are associated with alcohol consumption or alcohol-related harms, including alcohol consumption,^13,23,24,25,26,36,37,38^ age,^13,24,25,36,37^ gender^11,12,13,24,25,26,36,37,38^ race and ethnicity,^11,12,13,23,24,36^ income,^13,23,37^ education^13,24,25,26^ political party affiliation (eg, Democrat),^13,24^ religiosity,^37^ and sexual orientation.^39,40,41^ Participants’ self-reported race and ethnicity were collected to characterize the sample and examine associations with policy support. Race and ethnicity categories included American Indian or Alaska Native; Asian; Black or African American; Hispanic, Latino, or Spanish; Middle Eastern or North African; Native Hawaiian or Other Pacific Islander; White; and another race or ethnicity (any identity participants self-described in a free-response box) or more than 1 race or ethnicity. Participants additionally responded to questions about behaviors related to drinking (eg, noticing and reading the current alcohol health warning or practicing a religion that discourages drinking). Finally, NORC provided information on the Census region where participants resided, given that both alcohol consumption and support for alcohol control policies vary across regions.^42,43,44^ The survey did not include attention checks. Survey items are presented in eTable 1 in Supplement 1.

### Statistical Analysis

Per the preregistration, analyses excluded participants who completed less than 90% of the survey (36 participants [3% of those eligible]) or who completed the survey implausibly quickly (ie, less than one-third of the median [IQR] completion time of 11.8 [8.2-18.6] minutes; 51 participants [5% of those eligible]). Missing data on policy support ranged from 0.1% to 0.3% and on demographics from 0.0% to 4.3%.

First, we estimated the proportion of participants who supported, were neutral to, or opposed each policy. Second, we examined the extent to which behavioral and demographic characteristics were associated with overall support for alcohol control policies. Using ordinary least squares, we regressed overall support on behavioral characteristics (alcohol consumption during the past 4 weeks, binge drinking during the past 4 weeks [defined as ≥4 drinks on an occasion for women and ≥5 drinks for men], noticing the current alcohol health warning, reading the current alcohol health warning, and practicing a religion that discourages alcohol consumption) and demographic characteristics (age, gender, sexual orientation, racial or ethnic identity, education, low household income [<185% of the Federal Poverty Level], political party affiliation, and Census region). We included all characteristics in the same model. All characteristics were preregistered except for binge drinking, noticing the current alcohol health warning, and reading the current alcohol health warning; these were added to provide additional insight on whether behavioral characteristics are associated with policy support. We also conducted sensitivity analyses to examine associations when including only preregistered characteristics. We confirmed no concerning multicollinearity (variance inflation factors <2.3) and that errors were approximately normally distributed (based on visual inspection) and not heteroskedastic (Breusch-Pagan test, *P* = .58). We used the models to calculate average differential effects (ADEs; ie, differences in estimated mean overall support between groups on the 1-5 scale). In exploratory (non–preregistered) analyses, we also examined whether participant characteristics were associated with support for each individual policy, using the same approach as for the primary analyses. Finally, we characterized support for each policy among the groups associated with overall support in regression analyses.

We conducted analyses from January to July 2025 in Stata version 19.5 (StataCorp). We used complete case analysis and a critical α = .05. We did not adjust *P* values for multiple comparisons. Analyses weighted observations using survey weights to account for the sampling design and represent the population of US adults aged 21 years or older who consume alcohol. NORC calculated the survey weights based on participants’ probability of being selected into the panel and into this study, recruitment nonresponse, survey nonresponse, and poststratification adjustment (eMethods in Supplement 1). The sample was weighted to population benchmarks for adults aged 21 years or older obtained from the February 2024 Current Population Surveys.

## Results

The final analytic sample included 1036 participants (eFigure in Supplement 1). Participants’ mean (SD) age was 49.4 (16.5) years. Of all participants, 524 (weighted percentage, 52%) were men; 138 (12%) identified as Hispanic, Latino, or Spanish; 115 (weighted percentage, 11%) identified as Black or African American; 681 (weighted percentage, 65%) identified as White; 455 (weighted percentage, 50%) had some college or less educational attainment; and 247 (weighted percentage, 28%) had low household income (Table 1).

**Table 1.** Characteristics of a Nationally Representative Sample of US Alcohol Consumers Aged 21 Years or Older, 2024

Characteristic	Participants, No./total No. (weighted %)^a^
Behavioral	
Consumed alcohol in the past 4 wks	
1-2 d per wk	535/1036 (50)
3-7 d per wk	501/1036 (50)
Binge drank in the past 4 wks	
0 times	410/1036 (36)
≥1 times	626/1036 (64)
Noticed the current alcohol health warning	
No	101/1036 (10)
Not sure	407/1036 (37)
Yes	528/1036 (52)
Read the current alcohol health warning in past 30 d	
No	727/1036 (70)
Yes	283/1036 (27)
Have not seen an alcohol container in past 30 d	26/1036 (3)
Practice a religion that discourages alcohol consumption	
Not at all actively	775/1030 (73)
Somewhat or very actively	255/1030 (27)
Demographic	
Age, y	
21-29	115/1036 (14)
30-44	342/1036 (30)
45-59	247/1036 (24)
≥60	332/1036 (32)
Gender	
Woman	509/1036 (47)
Man	524/1036 (52)
Nonbinary or another gender^b^	3/1036 (<1)
Sexual orientation	
Straight or heterosexual	911/1026 (88)
Gay, lesbian, or bisexual	115/1026 (12)
Racial or ethnic identity^c^	
American Indian or Alaska Native	10/1026 (1)
Asian	39/1026 (6)
Black or African American	115/1026 (11)
Hispanic, Latino, or Spanish	138/1026 (12)
Middle Eastern or North African	2 /1026(0.2)
Native Hawaiian or Other Pacific Islander	1/1026 (<1)
White	681/1026 (65)
Another race or ethnicity or ≥1 race or ethnicity^d^	40/1026 (4)
Education	
High school diploma or less	166/1026 (26)
Some college	289/1026 (24)
College graduate or associate degree	361/1026 (30)
Graduate degree	210/1026 (20)
Annual household income, % Federal Poverty Level	
0 to <185	247/991 (28)
≥185	744/991 (72)
Political party identification	
Republican	311/1027 (30)
Democrat	434/1027 (43)
Independent or other^e^	282/1027 (27)
Census region	
Northeast	151/1036 (19)
Midwest	282/1036 (21)
South	325/1036 (38)
West	278/1036 (23)

^a^ Analyses are weighted responses such that results statistically represent US alcohol consumers aged 21 years or older. Percentages may not sum to 100% due to rounding.

^b^ Other gender identity included any identities participants self-described in a free-response box.

^c^ Race and ethnicity data were self-reported by participants in the survey.

^d^ Other race and ethnicity included any identity participants self-described in a free-response box.

^e^ Other political party affiliation included any affiliation participants self-described in a free-response box.

Support was highest for the policy to require calorie content information on alcohol containers: 56% (95% CI, 52%-60%) supported this policy and only 8% (95% CI, 6%-11%) opposed (Figure). Likewise, more participants supported than opposed policies prohibiting alcohol advertisements on television when children are likely to be watching (52%; 95% CI, 48%-56%), requiring drinks per container information (51%; 95% CI, 47%-55%), and requiring cancer warnings (49%; 95% CI, 45%-53%); opposition to these policies ranged from 10% (95% CI, 8%-13%) to 19% (95% CI, 16%-23%). By contrast, fewer people supported than opposed policies to lower the BAC limit for driving, prohibit alcohol sales late at night, and increase alcohol taxes (range supporting: 16% [95% CI, 13%-19%] to 25% [95% CI, 22%-29%]; range opposing: 43% [95% CI, 39%-47%] to 61% [95% CI, 57%-65%]). Support was lowest for the policy to reduce the number of outlets licensed to sell alcohol (10% [95% CI, 8%-13%] supported and 60% [95% CI, 56%-64%] opposed).

**Figure.**
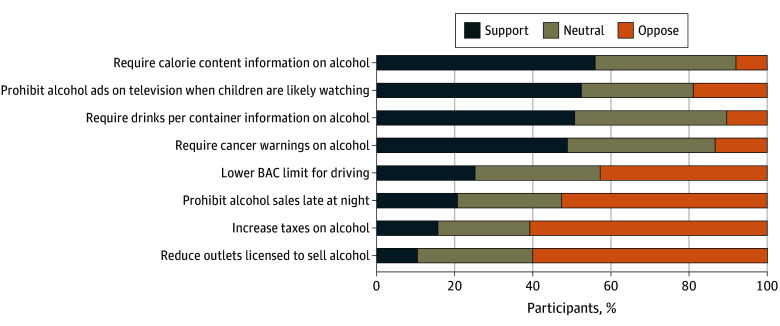
Support for 8 Alcohol Control Policies Among 1036 US Alcohol Consumers Aged 21 Years or Older, 2024 BAC indicates blood alcohol content.

The mean (SD) level of overall support for alcohol control policies (averaged across all policies) was 2.99 (0.67), close to the neither oppose nor support value of 3 on the 1 to 5 response scale. Several behavioral characteristics were associated with overall support (Table 2). Adults who drank more often (3-7 days per week) reported lower overall support than those who drank less often (1-2 days per week; ADE = −0.12; 95% CI, −0.23 to −0.02; *P* = .02), as did those who binge drank in the past month compared with those who did not (ADE = −0.15; 95% CI, −0.26 to −0.04; *P* = .01). These associations appeared to be due to lower support for increasing alcohol taxes, lowering the BAC limit for driving, and prohibiting alcohol sales late at night among those who drank more often or binge drank (Table 3). Adults who reported reading the current alcohol warning in the past 30 days reported higher overall support than those who reported not reading the warning or not seeing an alcohol container in the past 30 days (ADE = 0.14; 95% CI, 0.01 to 0.28; *P* = .04), due to their higher support for lowering the BAC limit, prohibiting alcohol sales late at night, increasing alcohol taxes, and reducing the number of outlets licensed to sell alcohol (Table 3).

**Table 2.** Associations of Behavioral and Demographic Characteristics With Overall Support for Alcohol Control Policies Among 982 US Alcohol Consumers Aged 21 Years or Older, 2024^a^

Characteristic	Support for overall alcohol control policies, ADE (95% CI)	*P* value
Behavioral		
Consumed alcohol in the past 4 wks		
1-2 d per wk	[Reference]	NA
3-7 d per wk	−0.12 (−0.23 to −0.02)	.02
Binge drank in the past 4 wks		
0 times	[Reference]	NA
≥1 times	−0.15 (−.26 to −0.04)	.01
Noticed the current alcohol health warning		
No or not sure	[Reference]	NA
Yes	−0.06 (−0.17 to 0.05)	.26
Read the current alcohol health warning in past 30 d		
No or have not seen an alcohol container in past 30 d	[Reference]	NA
Yes	0.14 (0.01 to 0.28)	.04
Practice a religion that discourages alcohol consumption		
Not at all actively	[Reference]	NA
Somewhat or very actively	0.08 (−0.06 to 0.21)	.27
Demographic		
Age, y		
21-34	[Reference]	NA
35-49	−0.09 (−0.24 to 0.06)	.22
50-64	−0.09 (−0.24 to 0.05)	.22
≥65	−0.07 (−0.23 to 0.10)	.41
Gender^b^		
Man	[Reference]	NA
Woman	0.22 (0.11 to 0.32)	<.001
Sexual orientation		
Straight or heterosexual	[Reference]	NA
Gay, lesbian, or bisexual	−0.08 (−0.27 to 0.10)	.39
Racial or ethnic identity^c^		
Asian	0.23 (−0.09 to 0.56)	.16
Black or African American	−0.11 (−0.31 to 0.09)	.27
Hispanic, Latino, or Spanish	0.16 (0.001 to 0.33)	.048
White	[Reference]	NA
Another race or ethnicity or >1 race or ethnicity^d^	0.07 (−.23 to 0.37)	.65
Education		
Some college or less	[Reference]	NA
College graduate or associate degree	0.08 (−0.03 to 0.19)	.17
Annual household income, % Federal Poverty Level		
0 to <185	[Reference]	NA
≥185	.02 (−0.11 to 0.15)	.73
Political party affiliation		
Republican	[Reference]	NA
Democrat	0.14 (0.01 to 0.27)	.03
Independent or other^e^	0.16 (0.02 to 0.29)	.03
Census region		
Northeast	[Reference]	NA
Midwest	−0.06 (−0.22 to 0.11)	.50
South	−0.08 (−0.24 to 0.09)	.35
West	0.04 (−0.13 to 0.22)	.62

Abbreviations: ADE, average differential effect; NA, not applicable.

^a^ Table presents ADEs (ie, differences in estimated means between groups) and 95% CIs from a multiple regression model including all variables shown in the Table. Analyses are weighted responses such that results statistically represent US alcohol consumers aged 21 years or older.

^b^ Individuals who identified as nonbinary or another gender were excluded due to small cell size.

^c^ Race and ethnicity data were self-reported by participants in the survey.

^d^ Other race and ethnicity included any identity participants self-described in a free-response box.

^e^ Other political party affiliation included any affiliation participants self-described in a free-response box.

**Table 3.** Associations of Behavioral and Demographic Characteristics With Support for Specific Alcohol Control Policies Among 984 US Alcohol Consumers Aged 21 Years or Older, 2024^a^

Characteristic	Policy support, ADE (SE)							
	Require calorie content on containers	Prohibit ads on TV when children likely watching	Require drinks per container information on containers	Require cancer warnings on containers	Lower BAC limit for driving	Prohibit alcohol sales late at night	Increase alcohol taxes	Reduce outlets licensed to sell alcohol
Behavioral								
Alcohol consumption during past 4 wks								
1-2 d per wk	[Reference]	[Reference]	[Reference]	[Reference]	[Reference]	[Reference]	[Reference]	[Reference]
3-7 d per wk	−0.02 (0.07)	−0.16 (0.10)	−0.11 (0.08)	−0.14 (0.09)	−0.05 (0.10)	−0.05 (0.10)	−0.36 (0.10)^b^	−0.12 (0.09)
Binge drinking during past 4 weeks								
0 times	[Reference]	[Reference]	[Reference]	[Reference]	[Reference]	[Reference]	[Reference]	[Reference]
≥1 times	−0.02 (0.08)	−0.18 (0.10)	−0.05 (0.08)	−0.07 (0.09)	−0.40 (0.11)^b^	−0.28 (0.10)^b^	−0.02 (0.10)	−0.17 (0.09)
Noticed the current alcohol health warning								
No or not sure	[Reference]	[Reference]	[Reference]	[Reference]	[Reference]	[Reference]	[Reference]	[Reference]
Yes	0.05 (0.08)	−0.16 (0.09)	0.03 (0.08)	0.00 (0.09)	−0.01 (0.10)	−0.20 (0.10)^b^	−0.15 (0.10)	−0.06 (0.08)
Read the current alcohol health warning in past 30 d								
No or have not seen an alcohol container in past 30 d	[Reference]	[Reference]	[Reference]	[Reference]	[Reference]	[Reference]	[Reference]	[Reference]
Yes	−0.04 (0.09)	0.02 (0.11)	0.02 (0.09)	−0.12 (0.11)	0.26 (0.12)^b^	0.38 (0.12)^b^	0.39 (0.12)^b^	0.25 (0.10)^b^
Practice of a religion that discourages alcohol consumption								
Not at all actively	[Reference]	[Reference]	[Reference]	[Reference]	[Reference]	[Reference]	[Reference]	[Reference]
Somewhat or very actively	−0.04 (0.09)	0.07 (0.12)	−0.01 (0.10)	0.10 (0.11)	0.02 (0.13)	0.27 (0.13)^b^	0.06 (0.12)	0.16 (0.11)
Demographic								
Age, y								
21-34	[Reference]	[Reference]	[Reference]	[Reference]	[Reference]	[Reference]	[Reference]	[Reference]
35-49	−0.09 (0.10)	−0.03 (0.13)	−0.13 (0.11)	−0.17 (0.13)	−0.18 (0.14)	−0.03 (0.13)	−0.06 (0.13)	−0.06 (0.13)
50-64	−0.14 (0.10)	−0.12 (0.13)	−0.20 (0.11)	−0.10 (0.14)	−0.18 (0.15)	0.16 (0.14)	−0.11 (0.14)	−0.04 (0.13)
≥65	−0.16 (0.12)	0.16 (0.14)	−0.20 (0.12)	−0.18 (0.15)	−0.48 (0.16)^b^	0.41 (0.15)^b^	−0.05 (0.15)	−0.05 (0.14)
Gender^c^								
Man	[Reference]	[Reference]	[Reference]	[Reference]	[Reference]	[Reference]	[Reference]	[Reference]
Woman	0.25 (0.07)^b^	0.34 (0.09)^b^	0.16 (0.08)^b^	0.28 (0.09)^b^	−0.10 (0.10)	0.42 (0.09)^b^	0.09 (0.09)	0.28 (0.09)^b^
Sexual orientation								
Straight or heterosexual	[Reference]	[Reference]	[Reference]	[Reference]	[Reference]	[Reference]	[Reference]	[Reference]
Gay, lesbian, or bisexual	−0.10 (0.14)	0.13 (0.16)	−0.12 (0.14)	−0.14 (0.17)	−0.09 (0.18)	−0.16 (0.15)	0.01 (0.16)	−0.16 (0.12)
Racial or ethnic identity								
Asian	0.34 (0.24)	0.25 (0.25)	0.25 (0.17)	0.41 (0.25)	−0.07 (0.28)	0.14 (0.34)	0.39 (0.27)	0.15 (0.24)
Black or African American	−0.22 (0.13)	−0.28 (0.17)	−0.18 (0.14)	−0.15 (0.17)	−0.10 (0.16)	−0.15 (0.16)	−0.17 (0.16)	0.36 (0.16)^b^
Hispanic, Latino, or Spanish	0.04 (0.12)	0.02 (0.15)	0.24 (0.13)	0.35 (0.13)^b^	0.00 (0.16)	0.13 (0.16)	0.14 (0.15)	0.38 (0.15)^b^
White	[Reference]	[Reference]	[Reference]	[Reference]	[Reference]	[Reference]	[Reference]	[Reference]
Another race or ethnicity or >1 race or ethnicity	−0.07 (0.17)	0.10 (0.21)	0.02 (0.23)	0.13 (0.20)	−0.30 (0.24)	0.09 (0.25)	0.26 (0.26)	0.33 (0.26)
Education								
Some college or less	[Reference]	[Reference]	[Reference]	[Reference]	[Reference]	[Reference]	[Reference]	[Reference]
College graduate or associate degree	0.16 (0.08)^b^	0.18 (0.10)	0.14 (0.08)	0.07 (0.09)	0.15 (0.11)	−0.03 (0.10)	0.01 (0.10)	−0.06 (0.09)
Annual household income, % FPL								
0 to <185	[Reference]	[Reference]	[Reference]	[Reference]	[Reference]	[Reference]	[Reference]	[Reference]
≥185	0.33 (0.10)	0.10 (0.11)	0.14 (0.09)	0.09 (0.10)	−0.09 (0.13)	−0.23 (0.12)	0.04 (0.12)	−0.18 (0.11)
Political party affiliation								
Republican	[Reference]	[Reference]	[Reference]	[Reference]	[Reference]	[Reference]	[Reference]	[Reference]
Democrat	0.13 (0.09)	0.11 (0.11)	0.29 (0.09)^b^	0.32 (0.11)^b^	−0.02 (0.12)	−0.06 (0.13)	0.35 (0.11)^b^	0.01 (0.11)
Independent or other	0.01 (0.09)	−0.02 (0.12)	0.13 (0.10)	0.30 (0.11)^b^	0.10 (0.13)	0.00 (0.13)	0.50 (0.12)^b^	0.22 (0.12)
Census region								
Northeast	[Reference]	[Reference]	[Reference]	[Reference]	[Reference]	[Reference]	[Reference]	[Reference]
Midwest	0.12 (0.12)	0.01 (0.15)	−0.14 (0.11)	−0.22 (0.13)	−0.06 (0.15)	−0.06 (0.14)	−0.06 (0.15)	−0.05 (0.12)
South	−0.03 (0.11)	−0.08 (0.15)	−0.25 (0.11)^b^	−0.32 (0.12)^b^	0.09 (0.14)	0.07 (0.15)	−0.12 (0.15)	0.03 (0.12)
West	0.15 (0.12)	0.10 (0.15)	−0.17 (0.12)	−0.12 (0.14)	0.16 (0.16)	0.06 (0.15)	0.05 (0.15)	0.13 (0.13)

Abbreviations: ADE, average differential effect; BAC; blood alcohol content; FPL, Federal Poverty Level.

^a^ Table presents ADEs (ie, differences in estimated means between groups) and SEs from a multiple regression model including all variables shown in the table. Missing data on policy support ranged from 0.0% to 0.2%. The policies analyzed were requiring alcoholic beverages to list the number of calories in the container; prohibiting alcohol advertisements on television when children are more likely to be watching; requiring alcoholic beverages to list the number of standard drinks in the container; requiring alcoholic beverages to display warnings that alcohol can cause cancer; lowering BAC at which people can legally drive; prohibiting stores, bars, and restaurants from selling alcohol late at night; increasing taxes on alcoholic beverages; and reducing the number of stores, bars, and restaurants that are licensed to sell alcohol in a given area. Analyses are weighted responses such that results statistically represent US alcohol consumers aged 21 years or older.

^b^ Statistically significant at *P* < .05.

^c^ Individuals who identified as nonbinary or another gender were excluded due to small cell size.

Demographic characteristics were also associated with overall support for alcohol control policies (Table 2). Women reported higher overall support than men (ADE = 0.22; 95% CI, 0.11-0.32; *P* < .001), a pattern that was consistent across most individual policies (Table 3). Adults who identified as Hispanic or Latino reported higher overall support than those who identified as non-Hispanic White (ADE = 0.16; 95% CI, 0.001-0.33; *P* = .048), due to higher support for requiring cancer warnings and reducing the number of outlets licensed to sell alcohol. Additionally, Democrats (ADE = 0.14; 95% CI, 0.01-0.27; *P* = .03) and political independents (ADE = 0.16; 95% CI, 0.02-0.29; *P* = .03) reported higher overall support than Republicans, due to higher support for requiring standard drink information and cancer warnings and for increasing alcohol taxes. Associations of behavioral and demographic characteristics with overall support were nearly identical in sensitivity analyses examining only preregistered characteristics (eTable 2 in Supplement 1).

Despite the differences in overall support by some characteristics, the large majority of participants—regardless of their characteristics—supported or were neutral toward policies to require calorie content information, drinks per container information, or cancer warnings on alcohol and to prohibit alcohol advertisements on television when children are likely to be watching; eTables 3-8 in Supplement 1 show support for each policy by subgroup. Moreover, few participants, regardless of subgroup, opposed these policies. For example, although overall support was lower among Republican participants, 40% (95% CI, 33%-47%) to 55% (95% CI, 48%-63%) supported requiring calorie content information, drinks per container, and cancer warnings on alcohol containers and few (8% [95% CI, 5%-12%] to 18% [95% CI, 13%-23%]) opposed these policies (eTable 8 in Supplement 1).

## Discussion

In this survey study of a nationally representative sample of US adults who consume alcohol, very few adults opposed policies to restrict advertising to children and require calorie content information, drinks per container information, and cancer warnings on alcohol containers. By contrast, a slight majority opposed policies to prohibit late-night alcohol sales, increase alcohol taxes, and reduce the number of outlets licensed to sell alcohol. This pattern was consistent across population groups, although overall support for alcohol control policies varied somewhat by behavioral and demographic characteristics such as usual alcohol consumption, gender, and political party identification. Together, these results suggest that many alcohol control policies would be well-received—or at least not strongly opposed—by US adults who consume alcohol.

Among alcohol consumers in our study, support was highest for policies that would restrict marketing to children and require more information on alcohol containers (eg, calorie content and cancer warnings). Prior research from both the US and other countries has similarly found high support for policies to require more information on alcohol containers^23,24,26,36,37,45,46^ and protect children from exposure to alcohol or alcohol advertising.^25,26,46^ Support was lower—especially among heavier drinkers and binge drinkers—for policies that would restrict or penalize drinking, such as increasing alcohol taxes or restricting the times or places where alcohol can be sold, also similar to prior studies.^26,36,37,46,47,48^ These findings are consistent with previous literature showing that support for policies depends in part on their perceived coerciveness or intrusiveness.^49,50,51,52,53^ Specifically, information provision policies (sometimes called pull policies) tend to garner greater public support than regulations and taxes (push policies),^49,50,51^ perhaps because people perceive the former to have lower financial or behavioral costs.^37,49,51,54^

Given that the regulatory and taxation policies we evaluated are considered highly cost-effective by the World Health Organization,^2,3,4^ it is perhaps disappointing that support for these policies was not higher. However, moderately high support and limited opposition to information provision policies suggests that these policies could offer a valuable starting point for addressing alcohol-related harms. In turn, support for more restrictive policies may grow after passage of ground softening information provision policies.^52^ For example, implementation of enhanced alcohol warnings in the Yukon, Canada, was associated with increased knowledge that alcohol can cause cancer,^55^ which in turn was associated with higher support for policies to increase the price of alcohol.^56^

The low opposition toward labeling policies is especially promising given the growing momentum for these policies in the US. In early 2025, the US Surgeon General called for policies to require cancer warnings on alcohol,^6^ and the US Alcohol and Tobacco Tax and Trade Bureau (which oversees labeling for most alcohol sold in the US) proposed requiring calorie content information be displayed on alcohol containers.^57^ Support for these policies was high in our study; approximately one-half or more of participants indicated they supported policies to require calorie content information, drinks per container information, and cancer warnings to appear on alcohol containers, and only 8% to 13% indicated opposition. Prior research spanning several decades and countries has also found high support for alcohol labeling policies,^23,24,25,37,48,58^ suggesting a growing consensus that policymakers and regulators can pursue labeling policies with less likelihood of opposition.

Support for alcohol control policies, especially for more restrictive policies like increasing taxes and restricting alcohol sales late at night, was lower among participants who reported drinking more often and those who reported binge drinking, consistent with prior research.^24,25,26,27^ This finding likely reflects that heavier drinkers would be most affected by these policies, and thus are less likely to support them. By contrast, support for these policies was higher among participants who reported having read the current alcohol health warning in the past 30 days; these adults may be more informed about alcohol’s harms and thus more willing to support alcohol control policies. Indeed, prior studies have found that people who are aware that alcohol causes harms such as cancer are more likely to support alcohol control policies.^24,26,59^ Alternatively, it is also possible that people who are more supportive of alcohol control policies are more likely to seek information about alcohol’s harms, including by reading warning labels.

When considering gender, racial and ethnic identity, and political party identification, overall support for alcohol control policies in our study generally mirrored patterns found in prior studies.^24,25,26,37^ For example, support was higher among women compared with men and people who identified as Democrats or political independents compared with Republicans. These differences suggest that social, cultural, and ideological factors may shape attitudes toward alcohol control policies. For example, higher support among Democrats and independents may reflect broader ideological orientations toward government regulation of health behaviors. Advocates and policymakers wishing to garner support for alcohol control policies may want to tailor messages to be relevant to specific population groups, particularly those that may be more skeptical of these policies. For example, research examining public support for obesity prevention policies suggests that messages about military readiness might be most effective for boosting support among conservatives, while messages about the health effects and health care costs of obesity may resonate most with liberals.^35^

Census region, age, sexual orientation, education, income, and practicing a religion that discourages alcohol consumption were not associated with overall support. While some of these associations differed from previous studies, perhaps due to differences in sample composition or timing, they suggest that policymakers can pursue alcohol control policies with less fear that these policies could alienate certain groups of constituents, such as young voters.

### Strengths and Limitations

Strengths of this study include the recruitment of a nationally representative sample of US adults who consume alcohol and examination of a broad range of evidence-based and timely alcohol control policies. Limitations include not assessing support for other promising alcohol control policies such as minimum unit pricing or more comprehensive advertising restrictions. Additionally, our sample included only alcohol consumers; assessing support also among nonconsumers is important given that alcohol control policies could prevent consumption among nonconsumers. Our findings, coupled with prior literature,^13,23,24,25,26,36,37,38^ suggest that support for alcohol control policies would likely be even higher among non-consumers.

## Conclusions

In this survey study of US adults who consume alcohol, many participants supported policies to protect children from alcohol advertising and to require more information on alcohol containers. Support was lower for increased taxes and restrictions on the times and places alcohol can be sold. Policymakers interested in alcohol control policies may wish to pursue advertising and information provision policies as a ground-softening strategy to build support for other types of policies.

## Notes

Supplement 1. eMethods. eTable 1. Survey measures eFigure. Participant flow diagram eTable 2. Associations of pre-registered behavioral and demographic characteristics with overall support for alcohol control policies among US alcohol consumers aged 21+ years, 2024 (n=982) eTable 3. Support for alcohol control policies by usual alcohol consumption during the past 4 weeks, n=1,036 US alcohol consumers aged 21+ years, 2024 eTable 4. Support for alcohol control policies by binge drinking during the past 4 weeks, n=1,036 US alcohol consumers aged 21+ years, 2024 eTable 5. Support for alcohol control policies by reading the current alcohol health warning in the past 30 days, n=1,036 US alcohol consumers aged 21+ years, 2024 eTable 6. Support for alcohol control policies by gender, n=1,033 US alcohol consumers aged 21+ years, 2024 eTable 7. Support for alcohol control policies by race/ethnicity, n=1,036 US alcohol consumers aged 21+ years, 2024 eTable 8. Support for alcohol control policies by political party affiliation, n=1,036 US alcohol consumers aged 21+ years, 2024 eReferences.

Supplement 2. Data Sharing Statement
