# Supplementary material for: Support for Alcohol Control Policies Among US Alcohol Consumers
Source: JAMA Netw Open. 2025 Oct 3;8(10):e2535337. doi: 10.1001/jamanetworkopen.2025.35337 (PMC12495495; doi:10.1001/jamanetworkopen.2025.35337)
Supplement: Supplement 1. — eMethods. eTable 1. Survey measures eFigure. Participant flow diagram eTable 2. Associations of pre-registered behavioral and demographic characteristics with overall support for alcohol control policies among US alcohol consumers aged 21+ years, 2024 (n=982) eTable 3. Support for alcohol control policies by usual alcohol consumption during the past 4 weeks, n=1,036 US alcohol consumers aged 21+ years, 2024 eTable 4. Support for alcohol control policies by binge drinking during the past 4 weeks, n=1,036 US alcohol consumers aged 21+ years, 2024 eTable 5. Support for alcohol control policies by reading the current alcohol health warning in the past 30 days, n=1,036 US alcohol consumers aged 21+ years, 2024 eTable 6. Support for alcohol control policies by gender, n=1,033 US alcohol consumers aged 21+ years, 2024 eTable 7. Support for alcohol control policies by race/ethnicity, n=1,036 US alcohol consumers aged 21+ years, 2024 eTable 8. Support for alcohol control policies by political party affiliation, n=1,036 US alcohol consumers aged 21+ years, 2024 eReferences. [file jamanetwopen-e2535337-s001.pdf]

Supplemental Online Content

Grummon AH, Chelius C, Lee CJY, et al. Support for alcohol control policies among alcohol consumers. *JAMA Netw Open*. 2025;8(10):e2535337.
doi:10.1001/jamanetworkopen.2025.35337

eMethods.

eTable 1. Survey measures

eFigure 1. Participant flow diagram

eTable 2. Associations of pre-registered behavioral and demographic characteristics with overall support for alcohol control policies among US alcohol consumers aged 21+ years, 2024 (n=982)

eTable 3. Support for alcohol control policies by usual alcohol consumption during the past 4 weeks, n=1,036 US alcohol consumers aged 21+ years, 2024

eTable 4. Support for alcohol control policies by binge drinking during the past 4 weeks, n=1,036 US alcohol consumers aged 21+ years, 2024

eTable 5. Support for alcohol control policies by reading the current alcohol health warning in the past 30 days, n=1,036 US alcohol consumers aged 21+ years, 2024

eTable 6. Support for alcohol control policies by gender, n=1,033 US alcohol consumers aged 21+ years, 2024

eTable 7. Support for alcohol control policies by race/ethnicity, n=1,036 US alcohol consumers aged 21+ years, 2024

eTable 8. Support for alcohol control policies by political party affiliation, n=1,036 US alcohol consumers aged 21+ years, 2024

eReferences.

This supplemental material has been provided by the authors to give readers additional information about their work.

© 2025 Grummon AH et al. *JAMA Network Open*.

eMethods

Recruitment through NORC AmeriSpeak

The AmeriSpeak panel is a large, three-stage, complex, probability-based panel funded and operated by NORC at the University of Chicago. AmeriSpeak is designed to be representative of the US household population. US households are randomly selected with a known, non-zero probability for the NORC National Frame as well as address-based sample frame. Selected households are recruited by mail, telephone, and face-to-face by field interviewers. NORC's National Frame is designed to cover at least 97% of the US population, including all 50 states and the District of Columbia, by supplementing the US Postal Service Delivery Sequence File to better represent hard-to-reach rural households. A household is considered recruited or responded if at least one adult in the household joins the panel. NORC provides additional details on panel construction in their technical overview of the AmeriSpeak panel.[1]

NORC invited 12,500 panelists to the survey described in the present study. Of these, 2,658 completed screening questions, of whom 1,123 were eligible and 1,036 completed the survey and were included in analyses. The weighted household response rate (AAPOR RR3) for the survey was 26.2%. This response rate accounts for the panel recruitment rate, panel retention rate, and survey participation rate.

Survey Weights

NORC calculated survey weights for all participants. The final weight variable is a product of three weights. First, NORC calculated the AmeriSpeak panel weights, which account for a panelist's probability of being selected into the panel, panel recruitment nonresponse adjustments, and poststratification adjustments of the recruited panel to match population benchmarks. Second, NORC calculated study-specific base weights, which account for a panelist's selection into this specific study and the inverse of selection probabilities associated with sample selection from the panel. Third, NORC calculated study specific final weights, which are the final weights developed for all completed cases for this study. These weights adjust the base weights to address survey nonresponse and align the survey sample to specific population benchmarks. The sample was weighted to population benchmarks for age, sex, Census division, race/ethnicity, education, age x sex, age x race/ethnicity, and race/ethnicity x sex for adults aged 21+ obtained from the February 2024 Current Population Surveys. The final weights may be trimmed to reduce the influence of extreme weights on survey estimates.

© 2025 Grummon AH et al. *JAMA Network Open*.

eTable 1. Survey measures

Construct	Item	Response scale
	**Introduction and Eligibility**	
Age	How old are you?<br><br>**[page break]**	**[Open ended, restricted to only whole #s 0-115; force response]**
Sex on birth certificate	Please select the sex you were assigned at birth as listed on your birth certificate. This may or may not be the same as your gender.	1=Male<br>2=Female<br>99=Something else
Gender	What is your gender?<br><br>**[page break]**	1=Woman<br>2=Man<br>3=Nonbinary<br>4=Prefer to self-describe:_____
Frequency of drinking version	During the past 4 weeks, how often did you drink 1 or more drinks of an alcoholic beverage? Examples of alcoholic beverages include beer, wine, liquor, malt beverages, hard seltzer, and hard cider.<br><br>**[page break]**	7=7 days per week<br>5.5=5-6 days per week<br>3.5=3-4 days per week<br>1.5=1-2 days per week<br>0.5=Less than 1 day per week<br>0=Not at all in the past 4 weeks
Amount of drinking	During the past 4 weeks, on the days when you drank alcoholic beverages, about how many drinks did you usually have? Common measures of “a drink” are a 12-ounce beer, a 5-ounce glass of wine, or a drink with one shot of liquor.	**[Drop down menu]**<br>More than 20<br>20<br>19<br>…<br>2<br>1<br>Less than 1<br><br>[Note on Response option coding:<br>0=Less than 1<br>1=1<br>2=2 …<br>Etc.<br>20=20<br>21=More than 20]

© 2025 Grummon AH et al. *JAMA Network Open*.

Construct	Item	Response scale
Binge drinking	**[skip if reported did not drink in past 4 weeks]**<br><br>Considering all types of alcoholic beverages, how many times during the past 30 days did you have ([5 if male or other or missing, 4 if female based on *sex*]) or more drinks on an occasion?<br><br>**[page break]**	[Drop down with options from to 30 to 0]<br><br>[Note on Response option coding:<br>0=0<br>1=1<br>2=2<br>…<br>30=30]
	**Recall and Use of Current Warnings**	
Prompt	**Next, we will ask about health warning labels on alcohol containers. Alcohol containers include bottles and cans of alcoholic beverages.**	
Noticing of current warning	The last time you saw an alcohol container, did it have a health warning on it?<br><br>**[page break]**	0=No<br>1=Yes<br>2=Not sure
Prompt	**The US requires a health warning to appear on alcohol containers.**	
Read current warning	Have you read the health warning on an alcohol container in the last 30 days?	0=No<br>1=Yes<br>2=I haven't seen an alcohol container in the past 30 days
	**Policy Support**	
Prompt	**The next questions are about different policies. For each of the following policies, indicate how much you would support or oppose that policy.**<br><br>**[randomize order of policies]**	
Policy support – advertising restrictions	Prohibit alcohol advertisements on TV when children are more likely to be watching.	1=Strongly oppose<br>2=Somewhat oppose<br>3=Neither oppose nor support<br>4=Somewhat support<br>5=Strongly support
Policy support – taxes	Increase taxes on alcoholic beverages.	1=Strongly oppose<br>2=Somewhat oppose<br>3=Neither oppose nor support<br>4=Somewhat support<br>5=Strongly support

© 2025 Grummon AH et al. *JAMA Network Open*.

Construct	Item	Response scale
Policy support – restrict number of licenses	Reduce the number of stores, bars, and restaurants that are licensed to sell alcohol in a given area.	1=Strongly oppose<br>2=Somewhat oppose<br>3=Neither oppose nor support<br>4=Somewhat support<br>5=Strongly support
Policy support – restrict time of day	Prohibit stores, bars, and restaurants from selling alcohol late at night.	1=Strongly oppose<br>2=Somewhat oppose<br>3=Neither oppose nor support<br>4=Somewhat support<br>5=Strongly support
Policy support – lower BAC for driving	Lower the blood alcohol content (BAC) at which people can legally drive.	1=Strongly oppose<br>2=Somewhat oppose<br>3=Neither oppose nor support<br>4=Somewhat support<br>5=Strongly support
Policy support – cancer warnings	Require alcoholic beverages to display warnings that alcohol can cause cancer.	1=Strongly oppose<br>2=Somewhat oppose<br>3=Neither oppose nor support<br>4=Somewhat support<br>5=Strongly support
Policy support – list number of standard drinks	Require alcoholic beverages to list the number of standard drinks in the container.	1=Strongly oppose<br>2=Somewhat oppose<br>3=Neither oppose nor support<br>4=Somewhat support<br>5=Strongly support
Policy support – list calories	Require alcoholic beverages to list the number of calories in the container.	1=Strongly oppose<br>2=Somewhat oppose<br>3=Neither oppose nor support<br>4=Somewhat support<br>5=Strongly support
	**Demographics**	
Introduction to demographics	**We are asking the questions in the next section to better understand who completed our survey.**	

© 2025 Grummon AH et al. *JAMA Network Open*.

Construct	Item	Response scale
Race/ethnicity	What is your race or ethnicity? Check all that apply.	1=American Indian or Alaska Native<br>2=Asian<br>3=Black or African American<br>4=Hispanic, Latino, or Spanish<br>5=Middle Eastern or North African<br>6=Native Hawaiian or Other Pacific Islander<br>7=White<br>8=Another race or ethnicity (please specify____)
Education	What is the highest degree or level of school you have completed?	1=Less than high school<br>2=High school graduate (or GED)<br>3=Some college or technical school<br>4=Associate's degree<br>5=Bachelor's degree<br>6=Graduate or professional degree
Sexual orientation	The next question is about your sexual orientation. Do you consider yourself to be…<br><br>**[page break]**	1=Straight or heterosexual<br>2=Gay or lesbian<br>3=Bisexual<br>4=Prefer to self describe:___________
Religious practice	How actively do you practice a religion that discourages drinking alcohol?	1=Not at all actively<br>2=Somewhat actively<br>3=Very actively
Political party affiliation	Do you consider yourself a Democrat, a Republican, an Independent, or something else?	1=Democrat<br>2=Lean Democrat<br>3=Lean Republican<br>4=Republican<br>5=Independent<br>6=Something else (please describe): _____
Household size	How many people are in your household, including you?	# of people [restricted to 1-20, whole numbers]

© 2025 Grummon AH et al. *JAMA Network Open*.

Construct	Item	Response scale
Income	Which of the following categories best describes your total household income before taxes in the last 12 months?<br><br>**[page break]**	1=Less than $10,000<br>2=$10,000 to $14,999<br>3=$15,000 to $24,999<br>4=$25,000 to $34,999<br>5=$35,000 to $49,999<br>6=$50,000 to $74,999<br>7=$75,000 to $99,999<br>8=$100,000 to $149,999<br>9=$150,000 to $199,999<br>10=$200,000 or more
Income in Poverty Line %s	**[Ask only if item household size = 1]**<br>Which of the following categories best describes your total household income before taxes in the last 12 months?<br><br>**[page break]**	1=Less than $19,578<br>2=Between $19,578 and $27,861<br>3=Between $27,862 and $45,180<br>4=$45,181 or more
Income in Poverty Line %s	**[Ask only if household size = 2]**<br>Which of the following categories best describes your total household income before taxes in the last 12 months?<br><br>**[page break]**	1=Less than $26,572<br>2=Between $26,572 and $37,814<br>3=Between $37,815 and $61,320<br>4=$61,321 or more
Income in Poverty Line %s	**[Ask only if household size = 3]**<br>Which of the following categories best describes your total household income before taxes in the last 12 months?<br><br>**[page break]**	1=Less than $33,566<br>2=Between $33,566 and $47,767<br>3=Between $47,768 and $77,460<br>4=$77,461 or more
Income in Poverty Line %s	**[Ask only if household size = 4]**<br>Which of the following categories best describes your total household income before taxes in the last 12 months?<br><br>**[page break]**	1=Less than $40,560<br>2=Between $40,560 and $57,720<br>3=Between $57,721 and $93,600<br>4=$93,601 or more
Income in Poverty Line %s	**[Ask only if household size = 5]**<br>Which of the following categories best describes your total household income before taxes in the last 12 months?<br>**[page break]**	1=Less than $47,554<br>2=Between $47,554 and $67,673<br>3=Between $67,674 and $109,740<br>4=$109,741 or more
Income in Poverty Line %s	**[Ask only if household size = 6]**<br>Which of the following categories best describes your total household income before taxes in the last 12 months?<br>**[page break]**	1=Less than $54,548<br>2=Between $54,548 and $77,626<br>3=Between $77,627 and $125,880<br>4=$125,881 or more

© 2025 Grummon AH et al. *JAMA Network Open*.

Construct	Item	Response scale
Income in Poverty Line %s	**[Ask only if household size = 7]**<br>Which of the following categories best describes your total household income before taxes in the last 12 months?<br>**[page break]**	1=Less than $61,542<br>2=Between $61,542 and $87,579<br>3=Between $87,580 and $142,020<br>4=$142,021 or more
Income in Poverty Line %s	**[Ask only if household size = 8]**<br>Which of the following categories best describes your total household income before taxes in the last 12 months?<br>**[page break]**	1=Less than $68,536<br>2=Between $68,536 and $97,532<br>3=Between $97,533 and $158,160<br>4=$158,161 or more
Income in Poverty Line %s	**[Ask only if household size = 9]**<br>Which of the following categories best describes your total household income before taxes in the last 12 months?<br><br>**[page break]**	1=Less than $75,530<br>2=Between $75,530 and $107,485<br>3= Between $107,486 and $174,300<br>4=$174,301 or more
Income in Poverty Line %s	**[Ask only if household size = 10]**<br>Which of the following categories best describes your total household income before taxes in the last 12 months?<br><br>**[page break]**	1=Less than $82,524<br>2=Between $82,524 and $117,438<br>3=Between $117,439 and $190,440<br>4=$190,441 or more
Income in Poverty Line %s	**[Ask only if household size = 11]**<br>Which of the following categories best describes your total household income before taxes in the last 12 months?<br><br>**[page break]**	1=Less than $89,518<br>2=Between $89,518 and $127,391<br>3=Between $127,392 and $206,580<br>4=$206,581 or more
Income in Poverty Line %s	**[Ask only if household size = 12]**<br>Which of the following categories best describes your total household income before taxes in the last 12 months?<br><br>**[page break]**	1=Less than $96,512<br>2=Between $96,512 and $137,344<br>3=Between $137,345 and $222,720<br>4=$222,721 or more
Income in Poverty Line %s	**[Ask only if household size = 13]**<br>Which of the following categories best describes your total household income before taxes in the last 12 months?<br><br>**[page break]**	1=Less than $103,506<br>2=Between $103,506 and $147,297<br>3=Between $147,298 and $238,860<br>4=$238,861 or more
Income in Poverty Line %s	**[Ask only if household size = 14]**<br>Which of the following categories best describes your total household income before taxes in the last 12 months?<br><br>**[page break]**	1=Less than $110,500<br>2=Between $110,500 and $157,250<br>3=Between $157,251 and $255,000<br>4=$255,001 or more

© 2025 Grummon AH et al. *JAMA Network Open*.

Construct	Item	Response scale
Income in Poverty Line %s	**[Ask only if household size = 15]**<br>Which of the following categories best describes your total household income before taxes in the last 12 months?<br><br>**[page break]**	1=Less than $117,494<br>2=Between $117,494 and $167,203<br>3=Between $167,204 and $271,140<br>4=$271,141 or more
Income in Poverty Line %s	**[Ask only if household size =16]**<br>Which of the following categories best describes your total household income before taxes in the last 12 months?<br><br>**[page break]**	1=Less than $124,488<br>2=Between $124,488 and $177,156<br>3=Between $177,157 and $287,280<br>4=$287,281 or more
Income in Poverty Line %s	**[Ask only if household size = 17]**<br>Which of the following categories best describes your total household income before taxes in the last 12 months?<br><br>**[page break]**	1=Less than $131,482<br>2=Between $131,482 and $187,109<br>3=Between $187,110 and $303,420<br>4=$303,421 or more
Income in Poverty Line %s	**[Ask only if household size = 18]**<br>Which of the following categories best describes your total household income before taxes in the last 12 months?<br><br>**[page break]**	1=Less than $138,476<br>2=Between $138,476 and $197,062<br>3=Between $197,063 and $319,560<br>4=$319,561 or more
Income in Poverty Line %s	**[Ask only if household size = 19]**<br>Which of the following categories best describes your total household income before taxes in the last 12 months?<br><br>**[page break]**	1=Less than $145,470<br>2=Between $145,470 and $207,015<br>3=Between $207,016 and $335,700<br>4=$335,701 or more
Income in Poverty Line %s	**[Ask only if household size = 20]**<br>Which of the following categories best describes your total household income before taxes in the last 12 months?<br><br>**[page break]**	1=Less than $152,464<br>2=Between $152,464 and $216,968<br>3=Between $216,969 and $351,840<br>4=$351,841 or more

© 2025 Grummon AH et al. *JAMA Network Open*.

eFigure 1. Participant flow diagram

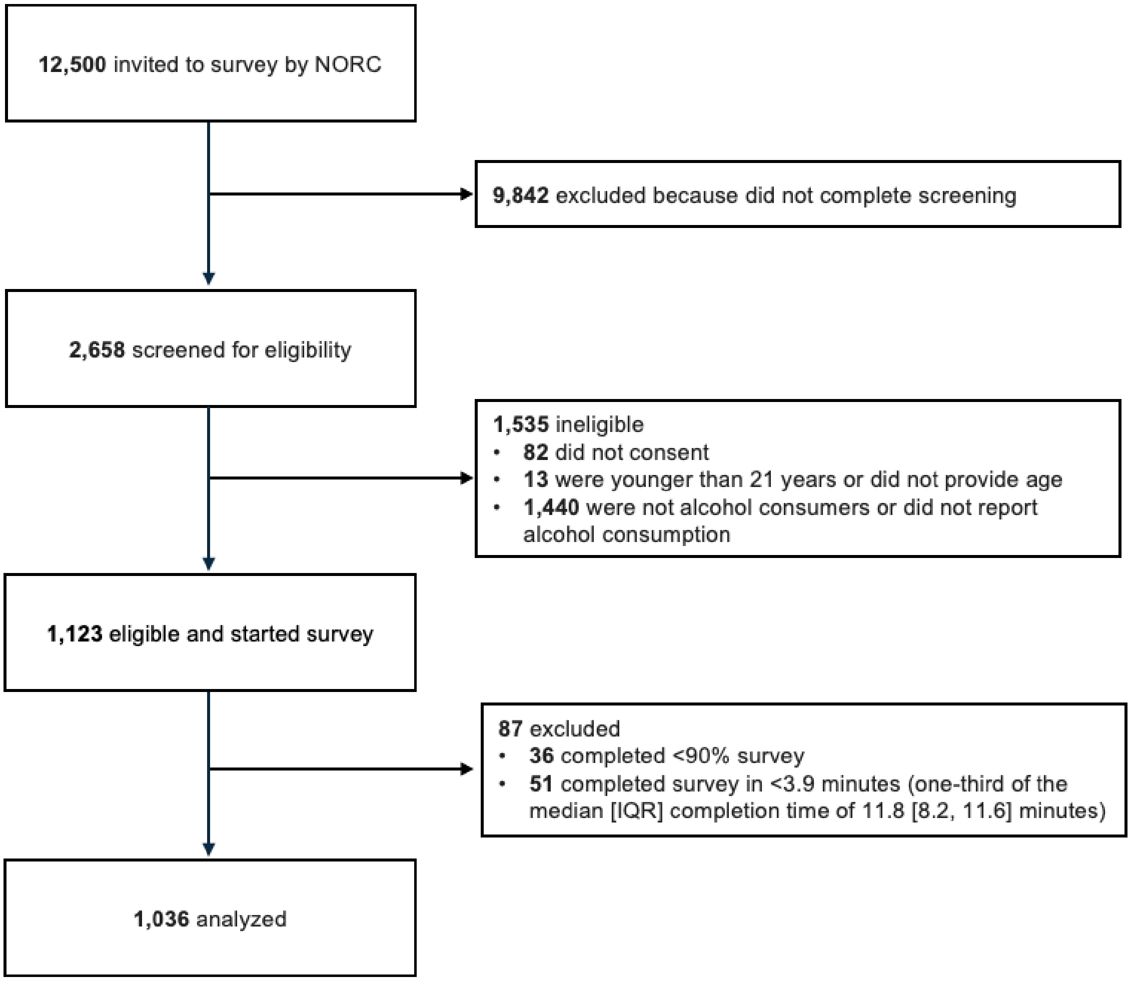

© 2025 Grummon AH et al. *JAMA Network Open*.

eTable 2. Associations of pre-registered behavioral and demographic characteristics with overall support for alcohol control policies among US alcohol consumers aged 21+ years, 2024 (*n*=982)

Characteristic	ADE	(95% CI)	*p*
**Behavioral**			
Alcohol consumption during past 4 weeks			
1-2 days per week	Ref		
3-7 days per week	**-0.15**	**(-0.25, -0.04)**	**.01**
Practice of a religion that discourages alcohol consumption			
Not at all actively	Ref		
Somewhat or very actively	0.07	(-0.07, 0.21)	.33
**Demographic**			
Age			
21-34 years	Ref		
35-49 years	-0.11	(-0.26, 0.04)	.16
50-64 years	-0.10	(-0.25, 0.04)	.17
65 years or older	-0.03	(-0.19, 0.14)	.73
Gender[a]			
Man	Ref		
Woman	**0.21**	**(0.11, 0.32)**	**<.001**
Sexual orientation			
Straight or heterosexual	Ref		
Gay, lesbian, or bisexual	-0.09	(-0.27, 0.09)	.35
Racial or ethnic identity			
White	Ref		
Asian	0.23	(-0.09, 0.55)	.16
Black or African American	-0.11	(-0.31, 0.09)	.30
Hispanic, Latino, or Spanish	0.16	(-0.01, 0.33)	.06
Another race or ethnicity or more than 1 race or ethnicity	0.08	(-0.22, 0.37)	.62
Education			
Some college or less	Ref		
College graduate or associate degree	0.09	(-0.02, 0.20)	.12
Household income, annual			
0% to <185% FPL	Ref		
≥185% FPL	0.03	(-0.10, 0.16)	.67
Political party affiliation			
Republican	Ref		
Democrat	**0.14**	**(0.01, 0.27)**	**.03**
Independent or other	**0.16**	**(0.02, 0.30)**	**.02**
Census region			
Northeast	Ref		
Midwest	-0.07	(-0.24, 0.10)	.41
South	-0.08	(-0.25, 0.09)	.34
West	0.03	(-0.14, 0.20)	.74

Abbreviations. ADE, average differential effect; CI, confidence interval.
Note. Table presents ADEs (i.e., differences in predicted means between groups) and 95% CIs from a multiple regression model including all variables shown in the table. Analyses weighted responses such that results statistically represent US alcohol consumers aged 21+ years.
[a]Individuals who identified as non-binary or another gender were excluded due to small cell size.

© 2025 Grummon AH et al. *JAMA Network Open*.

eTable 3. Support for alcohol control policies by usual alcohol consumption during the past 4 weeks among US alcohol consumers aged 21+ years, 2024 (*n*=1,036)

	Alcohol consumption during past 4 weeks	
**Policy**	**1-2 days per week**	**3-7 days per week**
Require calorie content information on alcohol		
Oppose	6%	10%
Neutral	36%	36%
Support	58%	54%
Prohibit alcohol ads on TV when children are likely watching		
Oppose	14%	24%
Neutral	31%	26%
Support	55%	49%
Requiring drinks per container information on alcohol		
Oppose	7%	14%
Neutral	39%	39%
Support	54%	47%
Require cancer warnings on alcohol		
Oppose	9%	17%
Neutral	40%	36%
Support	51%	47%
Lower Blood Alcohol Content (BAC) limit for driving		
Oppose	40%	45%
Neutral	32%	32%
Support	27%	23%
Prohibit alcohol sales late at night		
Oppose	52%	53%
Neutral	27%	26%
Support	21%	21%
Increase taxes on alcohol		
Oppose	57%	64%
Neutral	23%	24%
Support	20%	11%
Reduce outlets licensed to sell alcohol		
Oppose	57%	62%
Neutral	31%	29%
Support	12%	9%

Note. Analyses weighted responses such that results statistically represent US alcohol consumers aged 21+ years. Missing data ranged from 0.1% to 0.3%.

© 2025 Grummon AH et al. *JAMA Network Open*.

eTable 4. Support for alcohol control policies by binge drinking during the past 4 weeks among US alcohol consumers aged 21+ years, 2024 (*n*=1,036)

Policy	Binge drinking during past 4 weeks: 0 times	Binge drinking during past 4 weeks: 1 or more times
Require calorie content information on alcohol		
Oppose	6%	9%
Neutral	32%	38%
Support	62%	53%
Prohibit alcohol ads on TV when children are likely watching		
Oppose	12%	23%
Neutral	27%	29%
Support	61%	48%
Requiring drinks per container information on alcohol		
Oppose	8%	12%
Neutral	36%	41%
Support	56%	48%
Require cancer warnings on alcohol		
Oppose	11%	15%
Neutral	38%	38%
Support	51%	48%
Lower Blood Alcohol Content (BAC) limit for driving		
Oppose	38%	46%
Neutral	31%	33%
Support	31%	22%
Prohibit alcohol sales late at night		
Oppose	47%	56%
Neutral	29%	25%
Support	24%	19%
Increase taxes on alcohol		
Oppose	61%	60%
Neutral	20%	26%
Support	19%	14%
Reduce outlets licensed to sell alcohol		
Oppose	58%	61%
Neutral	35%	27%
Support	7%	12%

Note. Analyses weighted responses such that results statistically represent US alcohol consumers aged 21+ years. Missing data ranged from 0.1% to 0.3%.

© 2025 Grummon AH et al. *JAMA Network Open*.

eTable 5. Support for alcohol control policies by reading the current alcohol health warning in the past 30 days among US alcohol consumers aged 21+ years, 2024 (*n*=1,036)

Policy	Read the current alcohol health warning in past 30 days	
	No or have not seen alcohol container	Yes
Require calorie content information on alcohol		
Oppose	6%	13%
Neutral	36%	35%
Support	57%	52%
Prohibit alcohol ads on TV when children are likely watching		
Oppose	17%	25%
Neutral	30%	25%
Support	53%	50%
Requiring drinks per container information on alcohol		
Oppose	9%	15%
Neutral	41%	34%
Support	51%	51%
Require cancer warnings on alcohol		
Oppose	11%	20%
Neutral	39%	35%
Support	50%	45%
Lower Blood Alcohol Content (BAC) limit for driving		
Oppose	44%	39%
Neutral	33%	29%
Support	23%	32%
Prohibit alcohol sales late at night		
Oppose	54%	48%
Neutral	27%	25%
Support	19%	26%
Increase taxes on alcohol		
Oppose	63%	54%
Neutral	23%	26%
Support	14%	20%
Reduce outlets licensed to sell alcohol		
Oppose	62%	54%
Neutral	29%	32%
Support	9%	14%

Note. Analyses weighted responses such that results statistically represent US alcohol consumers aged 21+ years. Missing data ranged from 0.1% to 0.3%.

© 2025 Grummon AH et al. *JAMA Network Open*.

eTable 6. Support for alcohol control policies by gender among US alcohol consumers aged 21+ years, 2024 (*n*=1,033)

	Gender	
**Policy**	**Female**	**Male**
Require calorie content information on alcohol		
Oppose	5%	11%
Neutral	34%	37%
Support	60%	52%
Prohibit alcohol ads on TV when children are likely watching		
Oppose	14%	23%
Neutral	26%	31%
Support	59%	46%
Requiring drinks per container information on alcohol		
Oppose	9%	12%
Neutral	36%	41%
Support	55%	46%
Require cancer warnings on alcohol		
Oppose	10%	17%
Neutral	35%	41%
Support	55%	43%
Lower Blood Alcohol Content (BAC) limit for driving		
Oppose	45%	41%
Neutral	29%	35%
Support	26%	24%
Prohibit alcohol sales late at night		
Oppose	47%	58%
Neutral	26%	27%
Support	28%	15%
Increase taxes on alcohol		
Oppose	61%	61%
Neutral	24%	23%
Support	15%	16%
Reduce outlets licensed to sell alcohol		
Oppose	53%	66%
Neutral	34%	26%
Support	13%	8%

Note. Support among those who identified as nonbinary (*n*=3) are not reported due to small cell size. Analyses weighted responses such that results statistically represent US alcohol consumers aged 21+ years. Missing data ranged from 0.1% to 0.3%.

eTable 7. Support for alcohol control policies by race/ethnicity among US alcohol consumers aged 21+ years, 2024 (*n*=1,036)

	Race/ethnicity				
**Policy**	**Asian**	**Black**	**Hispanic**	**Other or multi-racial/ethnic**	**White**
Require calorie content information on alcohol					
Oppose	15%	14%	7%	24%	5%
Neutral	20%	41%	31%	26%	38%
Support	66%	45%	62%	50%	56%
Prohibit alcohol ads on TV when children are likely watching					
Oppose	22%	30%	18%	30%	16%
Neutral	12%	30%	31%	20%	30%
Support	66%	40%	50%	50%	54%
Requiring drinks per container information on alcohol					
Oppose	3%	23%	6%	32%	7%
Neutral	44%	33%	40%	21%	41%
Support	52%	44%	54%	47%	52%
Require cancer warnings on alcohol					
Oppose	14%	22%	6%	29%	12%
Neutral	22%	32%	32%	19%	43%
Support	64%	46%	62%	52%	45%
Lower Blood Alcohol Content (BAC) limit for driving					
Oppose	44%	40%	40%	51%	43%
Neutral	25%	41%	29%	28%	32%
Support	31%	19%	31%	21%	25%
Prohibit alcohol sales late at night					
Oppose	52%	48%	54%	44%	55%
Neutral	20%	36%	22%	21%	26%
Support	28%	16%	24%	35%	19%
Increase taxes on alcohol					
Oppose	53%	60%	52%	53%	64%
Neutral	10%	32%	30%	31%	21%
Support	37%	9%	17%	16%	15%
Reduce outlets licensed to sell alcohol					
Oppose	60%	45%	41%	59%	66%
Neutral	23%	39%	39%	26%	27%
Support	16%	16%	20%	15%	6%

Note. The other or multi category included those who identified as American Indian or Alaska Native, Middle Eastern or North African, Native Hawaiian or Other Pacific Islander, or another race or ethnicity not listed in the survey, or who identified as more than 1 race or ethnicity. Analyses weighted responses such that results statistically represent US alcohol consumers aged 21+ years. Missing data ranged from 1.0% to 1.2%.

eTable 8. Support for alcohol control policies by political party affiliation among US alcohol consumers aged 21+ years, 2024 (*n*=1,036)

Policy	Political Party Affiliation		
	Republican	Democrat	Independent
Require calorie content information on alcohol			
Oppose	8%	10%	5%
Neutral	37%	29%	47%
Support	55%	61%	48%
Prohibit alcohol ads on TV when children are likely watching			
Oppose	21%	19%	17%
Neutral	28%	25%	36%
Support	51%	57%	47%
Requiring drinks per container information on alcohol			
Oppose	12%	10%	9%
Neutral	46%	31%	45%
Support	43%	59%	46%
Require cancer warnings on alcohol			
Oppose	18%	14%	8%
Neutral	43%	30%	44%
Support	40%	56%	47%
Lower Blood Alcohol Content (BAC) limit for driving			
Oppose	42%	46%	38%
Neutral	33%	27%	39%
Support	25%	27%	23%
Prohibit alcohol sales late at night			
Oppose	52%	54%	52%
Neutral	23%	25%	32%
Support	25%	21%	16%
Increase taxes on alcohol			
Oppose	73%	59%	50%
Neutral	16%	23%	32%
Support	11%	18%	18%
Reduce outlets licensed to sell alcohol			
Oppose	63%	62%	53%
Neutral	26%	28%	37%
Support	11%	10%	11%

Note. Analyses weighted responses such that results statistically represent US alcohol consumers aged 21+ years. Missing data ranged from 1.0% to 1.2%.

eReferences

1. NORC. *Technical Overview of the AmeriSpeak Panel: NORC's Probability-Based Household Panel*. NORC; 2024. Accessed July 11, 2025. https://amerispeak.norc.org/content/dam/amerispeak/about-amerispeak/pdf/amerispeak-technical-overview.pdf

© 2025 Grummon AH et al. *JAMA Network Open*.
