# Supplementary material for: Support for Alcohol Control Policies Among US Alcohol Consumers
Source: JAMA Netw Open. 2025 Oct 3;8(10):e2535337. doi: 10.1001/jamanetworkopen.2025.35337 (PMC12495495; doi:10.1001/jamanetworkopen.2025.35337)
Supplement: Supplement 2. — Data Sharing Statement [file jamanetwopen-e2535337-s002.pdf]

Data Sharing Statement

Grummon. Support for Alcohol Control Policies Among US Alcohol Consumers. *JAMA Netw Open*. Published October 03, 2025. doi:10.1001/jamanetworkopen.2025.35337

Data
Data available: Yes
Data types: Deidentified participant data
How to access data: https://github.com/annagrummon/AlcoholControlPolicySupport_JNO2025
When available: With publication

Supporting Documents
Document types: Statistical/analytic code
How to access documents: The analytic code is available at: https://github.com/annagrummon/AlcoholControlPolicySupport_JNO2025.
When available: With publication

Additional Information
Who can access the data: Anyone can download the data.
Types of analyses: Any purpose.
Mechanisms of data availability: Publicly available to download.
Any additional restrictions: NA
